# Contribution of hypoxia inducible factor-1 during viral infections

**DOI:** 10.1080/21505594.2020.1836904

**Published:** 2020-10-31

**Authors:** Antonia Reyes, Nicolás Corrales, Nicolás M. S. Gálvez, Susan M. Bueno, Alexis M. Kalergis, Pablo A. González

**Affiliations:** a Millennium Institute on Immunology and Immunotherapy, Departamento de Genética Molecular y Microbiología, Facultad de Ciencias Biológicas, Pontificia Universidad Católica de Chile, Santiago, Chile; b Departamento De Endocrinología, Facultad De Medicina, Escuela De Medicina, Pontificia Universidad Católica De Chile, Santiago, Chile

**Keywords:** hypoxia, normoxia, RNA viruses, DNA viruses, virus life cycle, viral treatment

## Abstract

Hypoxia-inducible factor 1 (HIF-1) is a transcription factor that plays critical roles during the cellular response to hypoxia. Under normoxic conditions, its function is tightly regulated by the degradation of its alpha subunit (HIF-1α), which impairs the formation of an active heterodimer in the nucleus that otherwise regulates the expression of numerous genes. Importantly, HIF-1 participates in both cancer and infectious diseases unveiling new therapeutic targets for those ailments. Here, we discuss aspects related to the activation of HIF-1, the effects of this transcription factor over immune system components, as well as the involvement of HIF-1 activity in response to viral infections in humans. Although HIF-1 is currently being assessed in numerous clinical settings as a potential therapy for different diseases, up to date, there are no clinical studies evaluating the pharmacological modulation of this transcription factor as a possible new antiviral treatment. However, based on the available evidence, clinical trials targeting this molecule are likely to occur soon. In this review we discuss the role of HIF-1 in viral immunity, the modulation of HIF-1 by different types of viruses, as well as the effects of HIF-1 over their life cycle and the potential use of HIF-1 as a new target for the treatment of viral infections.

## Introduction

**Figure 1.**
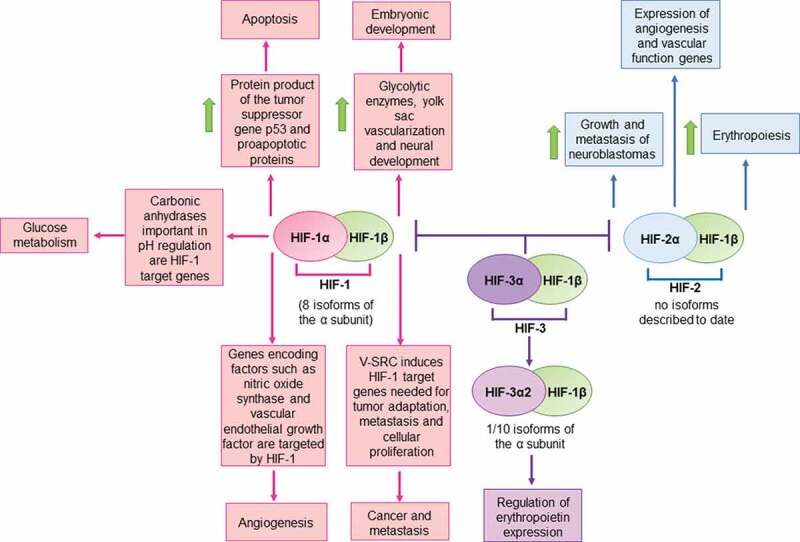
Schematic representation of HIF-regulated pathways

Hypoxia-inducible factor 1 (HIF-1) was described for the first time in 1992 as a critical regulator of oxygen tension levels in human hepatocellular carcinoma cells (Hep3B) [1]. Later, HIF-1 was characterized as a heterodimeric DNA-binding protein complex belonging to the PER-ARNT-SIM (PAS) family related to circadian rhythmicity in diverse organisms, voltage-activated potassium channels, and hydrogen sensing, among others [2]. HIF-1 consists of two polypeptide subunits with helix-loop-helix motifs/domains that are constitutively expressed in the cell [3]. This heterodimer is composed of an oxygen-regulated α subunit and a β subunit [4]. Currently, there are three known isoforms of the α subunit, namely HIF-1α, HIF-2α, and HIF-3α, with all three encoded in different loci and regulating distinct cellular functions, which give rise to three different proteins: HIF-1, HIF-2 and HIF-3, respectively [3, 5, 6]. Furthermore, the α subunits have different isoforms, with eight alternative splicing variants of the HIF-1α subunit, and 10 known isoforms of the HIF-3α subunit [7, 6]. Up to date, splice variants of the HIF-2α subunit have not been described. While HIF-1 mainly regulates glycolysis and angiogenesis, HIF-2 stimulates erythropoiesis [4], promotes the growth and metastasis of neuroblastomas, and activates the expression of target genes that modulate vascular function and angiogenesis in embryonic vascular endothelial cells, among other functions [5] Figure 1. On the other hand, the role of HIF-3 has remained somewhat poorly described. Interestingly, a new study has suggested a possible role for HIF-3α, isoform 2 as a transcriptional activator that regulates erythropoietin expression [8] Figure 1. Also, it has been described that HIF-3 may inhibit the activity of both, HIF-1 and HIF-2 [6] Figure 1. Under hypoxic conditions (<~1% O_2_), the HIF-1α subunit is activated and translocated to the nucleus, where it forms a heterodimer with the HIF-1β subunit and binds to the hypoxia response element (HRE) sequence in the regulatory regions of target genes. Combined with transcriptional coactivators recruited to neighboring sites, HIF-1 can induce the transcription of numerous genes [4,9].

HIF-1 is involved in embryonic development, apoptosis, glucose metabolism, angiogenesis and cancer metastasis, among others. HIF-2 is involved in the growth and metastasis of neuroblastomas, in the expression genes related to angiogenesis and vascular function, as well as erythropoiesis. The role of HIF-3 is less clear, but it has been reported to be involved in the regulation of erythropoietin expression and in the inhibition of activity of HIF-1 and HIF-2. Green arrows indicate upregulation of the elements indicated in the corresponding boxes.

Different studies have reported a variety of genes with different functions that are targeted by HIF-1. A set of genes induced by HIF-1 are involved in embryonic development [10, 11]. Importantly, a study reported that the loss of HIF-1α in embryonic stem cells (ESCs) led to a reduction in the expression of glycolytic enzymes, such as phosphoglycerate kinase 1 (PGK), lactate dehydrogenase A (LDH), aldolase A (ALDA) and glucose transporter 1 (GLUT-1). HIF-1α null embryos showed increased hypoxia and apoptosis, disorganized yolk sac vascularization, as well as abnormal neural development and cephalic vascularization [11] Figure 1. It has also been reported that transfection of cDNA encoding HIF-1α in murine ESCs promoted cardiogenesis, and that the activation of this transcription factor favored the differentiation and maturation of ESC-derived cardiomyocytes [10]. Other studies report a role for HIF-1 in tumor growth and cancer metastasis. For instance, V-SRC which is a transforming oncogene found in the Rous sarcoma virus, induces signal transduction pathways that elicit HIF-1, and HIF-1-regulated gene expression, such as vascular endothelial growth factor (VEGF) and enolase 1 (ENO1). Such factors may promote tumor adaptation to an hypoxic environment, which seem to be necessary for maintaining cellular proliferation, metastasis and reducing cell death [12, 13] Figure 1. HIF-1 has also been described to be involved in apoptosis. Indeed, HIF-1α has been described as capable of increasing and stabilizing the product of the tumor suppressor gene p53 and upregulating proapoptotic proteins, such as BNIP3 which has a promoter containing an HRE [14–16] Figure 1. Another process modulated by HIF-1 is angiogenesis. Genes that encode factors that are involved in different steps of this process have been described to be targeted by HIF-1, such as: nitric oxide synthase which participates in vasodilatation, vascular endothelial growth factor which increases vascular permeability, and collagen prolyl-4-hydroxylase that is involved in the degradation of the extracellular matrix during angiogenesis [17–20]. Figure 1. An additional process modulated by HIF-1 is glucose metabolism. Under hypoxic conditions, anaerobic glycolysis becomes activated, which induces lactate production and consequently a reduction in pH [21]. Importantly, transmembrane carbonic anhydrases, which play an important role in regulating pH have been shown to be HIF-1-target genes [22], among others Figure 1.

Importantly, a large amount of evidence suggests that HIF-1, and more specifically HIF-1α, plays a significant role in infectious and inflammatory diseases [23, 24]. For example, this transcription factor plays a role in the outcome of infections by different human pathogens, such as bacteria (e.g., *Staphylococcus aureus, Escherichia coli*, and *Acinetobacter baumannii*), fungi (e.g., *Tinea rubrum*), parasites (e.g., *Leishmania donovani*), and viruses (discussed in detail below) [25, 26, 24]. Numerous stimuli modulate this transcription factor’s expression during infections, which widely vary among pathogens [27]. There is also evidence showing that HIF-1α may be directly involved in the host response to pathogens through the modulation of immune cell functions, as we will further discuss in the following sections [28]. Considering all this, the transcription factor HIF-1 could be a potential target for novel therapies against some pathogens.

Here, we expand on previous studies and review articles that connect viral infections with HIF-1 [29, 27], by discussing additional viruses and recently-available literature related to HIF-1α and viral infections. Furthermore, we discusses the role of HIF-1 in the immune responses to viruses and potential new antiviral treatments based on HIF-1 modulation.

## Activation of HIF-1α

Both transcription and translation of HIF-1α mRNA are constitutive and are not affected by oxygen levels, overall remaining constant in the cell under normoxic (~21% O_2_) conditions [4,30]. However, the HIF-1α subunit has a half-life of approximately 5 minutes. Thus, due to its rapid degradation during normoxia, it is almost undetectable [4]. Under these conditions, the von Hippel-Lindau (pVHL) ubiquitin E3 ligase complex tags HIF-1α with ubiquitin leading to its rapid degradation [31]. Binding of pVHL with HIF-1α requires hydroxylation of two proline residue by the prolyl-hydroxylase domain proteins (PHDs). These proteins require oxygen, Fe^2+^, and ascorbate as cofactors for the hydroxylation process [4]. When oxygen levels decrease, the degradation of HIF-1α no longer occurs, as PHDs are not active, and no proline residues in pVHL are modified. This degradation abrogates the interaction between pVHL and HIF-1α. Lack of this interaction results in the stabilization of HIF-1α and its translocation from the cytoplasm to the nucleus [32,33]. Under these circumstances, HIF-1α dimerizes with HIF-1β in the nucleus to form the HIF-1α/β heterodimer or HIF-1, which is transcriptionally active [34, 30].

HIF-1α contains two transactivation domains: the N-terminal Transactivation Domain (N-TAD) and the C-Terminal Transactivation Domain (C-TAD). Under normoxic conditions, an asparagine residue in the C-TAD is hydroxylated by the asparagine hydroxylase factor inhibiting HIF-1 (FIH-1), which is also oxygen-dependent [3,4]. This hydroxylation prevents the recruitment of transcriptional coactivators that bind to this region of HIF-1α, such as CBP/p300 [4]. Under hypoxic conditions, the hydroxylation of this asparagine is suppressed, allowing the interaction between C-TAD and CBP/p300 [9].Interestingly, several viruses induce the degradation of PHDs as a mechanism to activate HIF-1α during infections, although there is also evidence indicating that some viruses may alternatively use other pathways to activate HIF-1α. For instance, infection with viruses such as hepatitis C or hepatitis B can promote processes that induce oxidative stress in the cell, influencing the stability of HIF-1α after infection [27]. Alternatively, other viruses such as Epstein-Barr virus may use intracellular kinases to induce the activation of HIF-1α, which is further discussed below [27].

## HIF-1α and immunity

The contribution of HIF-1 to some types of immune responses has been widely studied and extensively reviewed by Palazon *et al*. and Hellwig-Bürgel *et al*. [28,35]. This review focuses on the role of HIF-1 in viral infections and its role in the context of such immune responses, focusing on innate and adaptive immune components.

Innate immune components are responsible for immediate and early recognition of events after pathogen infection and are aimed to rapidly counteract their presence and replication [36]. As constant interactions occur between different types of immune cells, the innate immune response will likely influence the following adaptative immune response elicited by the host against the pathogen [37].

Previous studies have shown that inhibition of serine/threonine kinases and phosphatases can modulate HIF-1 activity, as treating cells with genistein -an inhibitor of tyrosine kinase- has significant effects on the transcriptional activity of HIF-1 [38, 39, 40]. However, the exact mechanisms regulating this activity are somewhat controversial due to contradictory reports on both positive and negative regulation of HIF-1 activity [41, 42]. Activation of the PI3/Akt pathway increases HIF-1α protein levels [43]. Consistently, inhibition of mTOR -and, therefore, inhibition of PI3/Akt- reduces the synthesis of HIF-1α [44, 45, 46]. Noteworthy, NF-κB is activated during hypoxia and involves the activation of IκB kinase-β in HeLa cells [47]. This activation inhibits neutrophil apoptosis via NF-κB, resulting in sustained inflammation [48]. Thus, inhibition of HIF-1α or PHDs could be used as a strategy for dampening exacerbated inflammatory responses. There is no evidence showing that MAPK levels affect the stabilization of HIF-1α, although a study reported that they might increase the trans-activation ability of HIF-1 [28].

Interferons are a group of cytokines that can either alert cells for limiting viral replication (mainly type-I interferons) or modulate immune system components (mainly type-II interferons) for virus control [49]. Under hypoxic conditions, the antiviral effects of IFN-α and IFN-γ are higher than in aerobic conditions, yet the mechanisms underlying these observations are unknown. However, it has been hypothesized that hypoxia may increase the expression of the corresponding IFN receptors, increasing signal transduction, or expression of IFN-stimulated genes (ISGs) [50].

Adaptive immunity is characterized as more specific than innate immunity and is involved in establishing immunological memory, yet this response takes some time to establish [51]. Professional antigen-presenting cells (APCs), such as dendritic cells (DCs), link innate and adaptive immunity, activating antigen-specific helper T cells that can help B cells differentiate and produce a particular antibody isotype [52]. These helper T cells can also activate cytotoxic T lymphocytes (CTLs) [49]. Noteworthy, the function and relevance of HIF-α over DC function is not fully understood; however, hypoxic conditions induce the production of numerous proinflammatory cytokines by these cells, such as TNF-α and IL-1β [53], and also modulates chemokine receptor expression [54]. In turn, these cytokines induce the expression of nitric oxide synthase (NOS), and the production of nitric oxide (NO), which may cause PHD inhibition by O_2_ competition, causing HIF-1α accumulation in normoxic conditions [28]. On the other hand, NO inhibition of active HIF-1 formation in hypoxic conditions may be explained by direct activation of PHDs via the redistribution of intracellular oxygen, given that NO inhibits mitochondrial O_2_ consumption [28].

In contrast to the effect of HIF-1α over DCs, this transcription factor may have suppressive effects on the production of proinflammatory cytokines by CD4^+^ and CD8^+^ T cells, such as IFN-γ. There might be a HIF-1-mediated anti-inflammatory response pathway in T cells, complementing immunosuppressive signaling mediated by extracellular adenosine produced by numerous cells, such as fibroblasts, epithelial cells, and muscle cells in different tissues [55, 56]. Noteworthy, hypoxia induces the expression of T cell activation-related receptors, such as 4–1BB and OX40, and inhibitory receptors such as LAG3 and CTLA-4, as well as effector molecules such as granzyme B [35].

Importantly, HIF-1α has been reported to have a vital role over the survival of T cell, the regulation of T and B cells, and the regulation of myeloid cells such as macrophages and neutrophils [57]. Consistently, HIF-1α plays a crucial role in the survival of T cells by preventing activation-induced cell death under hypoxic conditions [35]. This observation makes HIF1-α a key factor in regulating inflammatory processes.

HIF-1 activity has an impact on T cell activation and differentiation. For instance, HIF-1α is highly expressed in Th17-polarized T cells [35], and the deletion of this gene in T cells impairs the differentiation of CD4^+^ into this T-helper phenotype *in vivo* [58]. There is also evidence suggesting that HIF-1 negatively regulates Treg development [58], and in CD8^+^ T cells HIF-1 activation promotes glycolytic metabolism and effector functions, such as granzyme B expression [59].

## HIF-1 in viral infections

During viral infections, many studies report that HIF-1α is upregulated in infected cells, suggesting that this factor may have favorable effects for the virus, rather than for the host. Nevertheless, there is also evidence showing that some viruses downregulate the activity of this transcription factor. Altogether, the mechanisms by which viruses modulate HIF-1 seem quite variable and are discussed below in detail for different types of viruses.

### Double-stranded DNA viruses

#### Human papillomavirus

**Figure 2.**
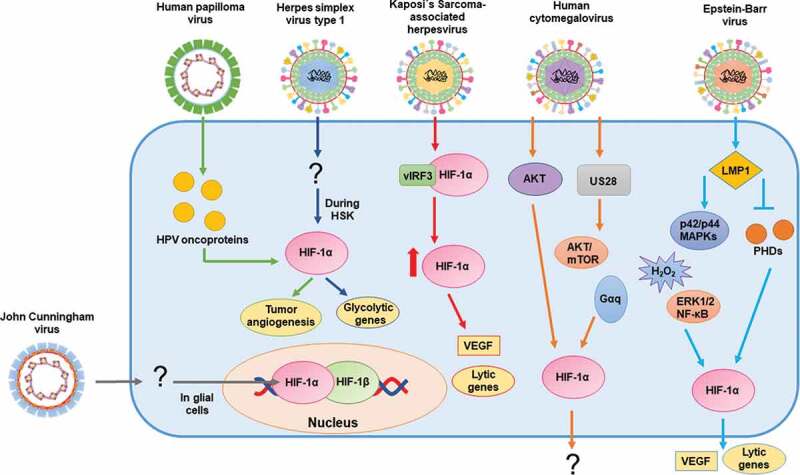
Schematic representation of the effects of double-stranded DNA viruses on HIF-1α and (possible) mechanisms of action

Human papillomaviruses (HPV) are known for producing cervical cancer, although they can also elicit other types of cancer [60]. Overexpression of HIF-1α has been reported to be a marker of poor prognosis in patients with cervical cancer. A study performed with 91 patients showed that individuals with higher levels of HIF-1α in tumor histological samples -obtained surgically- had significantly shorter overall survival rates and disease-free periods than those with lower levels [61]. This observation is consistent with human papillomavirus type 16 (HPV-16) oncoproteins showing an increased capacity to stabilize HIF-1α without necessarily increasing HIF-1α mRNA levels [62]. There is also evidence indicating that these oncoproteins -especially E6 and E7- promote the expression of vascular endothelial growth factor (VEGF) and other angiogenesis factors, such as IL-8, which can promote tumor angiogenesis via a HIF-1α/VEGF pathway in non-small cell lung cancers and human cervical carcinoma cells [63]. Furthermore, there is evidence showing that several HPVs, both low- and high-risk types, can enhance the expression of HIF-1α in human foreskin keratinocytes under hypoxic conditions by stabilizing HIF-1α. However, later studies showed that this increased activation of HIF-1α did not involve the PI3/mTOR, nor the VHL pathway as expected [64]. The information exposed above, suggest that HIF-1 modulation during HPV infection may be beneficial for the control of the disease Figure 2.

From left to right: John Cunningham virus (JCV) infection of glial cells increases the levels of HIF-1α in the nucleus; Human papilloma virus (HPV) viral oncoproteins stabilize HIF-1α; HIF-1α is stabilized in herpes stromal keratitis (HSK) by Herpes simplex virus type 1 (HSV-1) infection; Kaposi´s Sarcoma-associated herpesvirus (KSHV) upregulates HIF-1α transcription levels and stabilizes HIF-1α via its interaction with vIRF3; Human cytomegalovirus (HCMV) increases the expression and stabilization of HIF-1α; Epstein-Barr virus (EBV) induces the synthesis of HIF-1α protein and increases its mRNA levels via LMP1.

#### Epstein-Barr virus

Epstein-Barr virus (EBV, HHV-4) is a herpesvirus associated with Hodgkin’s lymphoma, B cell lymphoma, and nasopharyngeal carcinoma [65]. EBV is highly prevalent worldwide, with most infected individuals not showing clinical symptoms [65]. The latent membrane protein 1 (LMP1) of EBV induces the synthesis of the HIF-1α and promotes the expression of HIF-1 response-genes, such as VEGF [66, 68]. The mechanism of action underlying this activation in nasopharyngeal epithelial cells requires the up-regulation of Siah1 E3 ubiquitin ligase by LMP1. This up-regulation leads to proteasomal degradation of PHD1 and PHD3. These events promote the stabilization of HIF-1α by preventing the formation of the VHL/HIF-1α complex [66]. Another study suggests that LMP1 increases the expression of HIF-1α through the p42/44 MAPK pathway and H_2_O_2_ [68]. This study also indicates that the activity of the promoter of the HIF-1α gene is induced by the latent membrane protein 1 C-terminal activating region 1 recruited extracellular signal-regulated protein kinases 1 and 2/NF-κB pathway (LMP1 CTAR1-recruited ERK1/2/NF-κB pathway) [67]. Mechanistically, there is evidence showing that LMP1 also enhances mRNA levels of HIF-1α via reduced expression of the RNA-destabilizing proteins tristetraprolin (TTP) and pumilio RNA-binding family member 2 (PUM2). These proteins are RNA binding proteins (RBPs) that induce a decay of the HIF-1α mRNA by binding to AU rich elements in mRNAs and repressing their transcription, thus promoting mRNA degradation [67]. Moreover, HIF-1α plays a role in the induction of the lytic cycle of EBV, as hypoxic conditions increase the expression of the immediate-early protein Zta, which mediates the switch between latent and lytic infection, consequently increasing the number of viral DNA copies, as determined in a B-lymphoblastoid cell line [69]. Another study showed that HIF-1α binds to the promoter of the latent-lytic switch *BZLF1* gene Zp, activating its transcription and likely inducing EBV lytic-gene expression, suggesting EBV effects over HIF-1 and vice versa [70] Figure 2.

#### Kaposi’s sarcoma-associated herpesvirus

Kaposi’s Sarcoma-associated herpesvirus (KSHV, HHV-8) is the causative agent of Kaposi’s Sarcoma cancer, characterized by skin lesions with excessive vascularization of the epithelium [71]. Importantly, infections with KSHV have been reported to upregulate the transcript levels of both HIF-1α and HIF-2α in endothelial cells [72]. Noteworthy, KSHV viral homolog of the host interferon regulatory factor 3(IRF), vIRF3 interacts with HIF-1α to stabilize its protein levels and leads to its transcriptional activation [73]. This interaction also prevents the degradation of HIF-1α under normoxic conditions [73]. Kaposi’s sarcoma is a highly angiogenic endothelial tumor with VEGF playing an important role in the regulation of angiogenesis [74, 75]. The interaction between vIRF3 and HIF-1α induces VEGF expression and facilitates endothelial tube growth [73]. Pyruvate kinase-2 acts as a coactivator of HIF-1 and increases the levels of angiogenic factors, such as VEGF [76].

Hypoxia may have a role in the switch between latent to lytic infections of this virus. Interestingly, the KSHV latency-associated nuclear antigen (LANA) has been reported to associate with HIF-1α, enhancing the latter’s transcriptional activity and its mRNA levels. HIF-1α:LANA complex could bind to the HRE of the *Rta* promoter and upregulate lytic *Rta* gene expression when KSHV-infected cells are under hypoxic conditions [77]. Hypoxic conditions also induce lytic replication by this virus, via the viral inducer 12-O-tetradecanoylphorbol-13-acetate, that increases the level of IL-6 in HHV-8 infected cells. This increase stimulates spindle cell growth and plays a role in activating the production of angiogenic factors in infected cells [78]. The inhibition of KAP1 (KRAB-associated protein 1) enhances the association of HIF-1α and RBP-Jκ, leading to RTA expression, which has been shown to activate the expression of early and late genes in the KSHV lytic cycle [79]. Another study reported an effect of HIF-1α in KSHV infection as metabolic reprograming of KSHV-infected B cells under hypoxic conditions via vGPCR (G protein-coupled receptor), a lytic cycle-associated- and oncogenic protein that stimulates angiogenesis by increasing the secretion of VEGF [80] Figure 2.

#### Human cytomegalovirus

Human cytomegalovirus (HCMV or HHV-5) is also highly prevalent in the human population [81]. Although initially thought to be innocuous, more recent studies have shown multiple pathologies related to this virus, involving a wide range of tissues due to its broad cellular tropism [81]. Human fibroblasts have shown increased HIF-1α expression after infection with HCMV [82]. This effect was also seen when cells were inoculated with UV-inactivated HCMV, indicating that HIF-1α induction occurs due to the interaction of virus structural components with the cell and that viral gene expression is not necessary to induce this response [82]. Consistently, increased phosphorylation of the protein kinase AKT was reported, which is required for the induction of HIF-1α [82]. Interestingly, the HCMV-encoded chemokine receptor US28 increases the stabilization of HIF-1α via a Gq protein alpha subunit- (Gαq), calcium/calmodulin-dependent protein kinase II- (CaMKII) and AKT/mTOR-dependent manner, which supports the notion that HCMV infection may be favored by HIF-1 activation [83] Figure 2.

#### Herpes simplex virus type 1

Herpes simplex virus type 1 (HSV-1 or HHV-1) infection is lifelong, like other herpesviruses infections, and recurrent reactivations may occur throughout life. HSV-1 elicits several diseases, both mild and life-threatening. Importantly, HSV-1 can produce herpes stromal keratitis (HSK), which translates into corneal chronic inflammatory conditions and hypoxic conditions [84]. Importantly, HIF-1α stabilization has been observed in infiltrating immune cells during HSK, as well as increased expression of HIF-1 target genes, such as genes related to the glycolytic and lactate pathways in infected corneas [84]. Interestingly, depletion of neutrophils infiltrating the corneas significantly reduced the development of hypoxia in this tissue. This reduction may affect the development of HSK, given that neutrophils are considered essential for the development of this disease [84]. Although an inhibitor of HIF-1α reduced the severity of neovascularization in HSK, the corneas’ opacity was still observed [84]. Thus, the contribution of HIF-1α in HSK is not entirely understood Figure 2.

#### John Cunningham virus

The John Cunningham virus (JCV) has been reported to be the etiological cause of progressive multifocal leukoencephalopathy, a demyelinating disease similar to multiple sclerosis, but with a much quicker progressiveness [85]. Glial cells infected with JCV display increased levels of HIF-1α in the nucleus as compared to healthy glial cells. In this study, the presence of HIF-1α promoted the activation of the JCV early and late promoters and was shown to bind to the JCV control region, which may lead to the activation of the JCV promoter [86]. Thus, HIF-1α may be required by JCV for its replication cycle Figure 2.

### Positive-sense single-stranded RNA viruses

#### Hepatitis C virus

**Figure 3.**
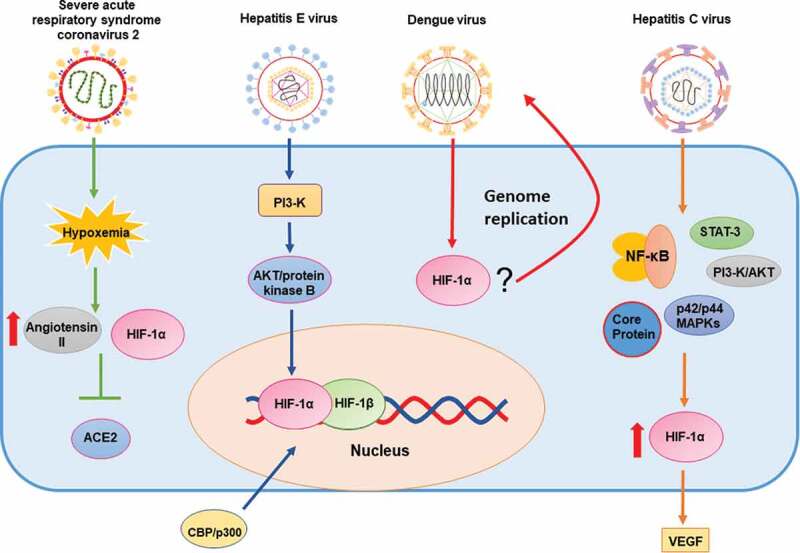
Schematic representation of the effects of positive single-stranded RNA viruses on HIF-1α and (possible) mechanisms of action

Hepatitis C virus (HCV) is a pathogen capable of causing severe liver diseases, such as cirrhosis or hepatocellular carcinoma in humans. Its infection is considered chronic if untreated, due to the ability of this virus to evade the host’s immune response for extended periods [87]. Importantly, low oxygen concentrations enhance hepatitis C virus replication and induce the upregulation of host genes related to hypoxic stress, such as those involved in glycolytic metabolism, cell growth, and proliferation [88]. Furthermore, HCV stabilizes HIF-1α by activating NF-κB, signal transducer and activator of transcription 3 (STAT-3), PI3-K/AKT, and p42/44 mitogen-activated protein kinase pathways. The induction of HIF-1α leads to the stimulation of VEGF [43]. A study reports that the induction of the HCV core protein leads to stabilization and overexpression of HIF-1α in a cancer cell line and, consequently, to the stimulation of VEGF [89]. HIF-1α stabilization was shown to be insensitive to antioxidant treatments, and mimicking an impairment of mitochondrial oxidative phosphorylation elicited the stabilization of this transcription factor [90]. Therefore, HCV stabilization of HIF-1α by different mechanisms may be contributing to increased HIF-1α upon viral infection with this virus Figure 3.

From left to right: Severe acute respiratory syndrome coronavirus 2 (SARS-CoV-2) causes hypoxemia. The accumulation of HIF-1α increase the levels of angiotensin II and decreases ACE2 expression; Hepatitis E virus (HEV) increases AKT/protein B and stabilizes HIF-1α; HIF-1α regulates genome replication of Dengue virus (DENV); Hepatitis C virus (HCV) stabilizes and promotes the overexpression of HIF-1α via NF-κB, STAT-3, PI3-K/AKT, p22/p44 MAPKs and the HCV core protein.

#### Hepatitis E virus

Hepatitis E virus (HEV) is another etiological agent producing severe liver disease. HEV has an incidence estimated at 20 million new infections every year [91]. Few studies have assessed HEV and HIF-1. One study showed that HEV stabilizes HIF-1α and increases its binding to HRE. This binding increases the transactivation activity of HIF-1 in cells expressing the viral protein ORF3 through the phosphatidylinositol-3-kinase (PI3K)-mediated activation of AKT/protein kinase B. The HIF-1 heterodimer also recruited CBP/p300 to particular gene promoters, in which cases the phosphorylation of CBP/p300 was required for the interaction [92] Figure 3.

#### Dengue virus

Dengue virus (DENV) is a *Flavivirus* transmitted by *Aedes* mosquitos in tropical and subtropical areas [93]. The infection of this virus leads to flu-like symptoms such as fever, joint pain, and a rash, but in some cases, it can lead to more severe pathologies and reach a mortality rate of up to 20% in some areas [93]. In DENV-infected cells, hypoxia was not shown to have a significant effect on virus entry and viral RNA translation but was shown to enhance DENV genome replication. Interestingly, the hypoxia-mediated enhancement of DENV replication was facilitated by HIF-1α/2α and by the serine/threonine kinase AKT, correlating with increased anaerobic glycolysis. Reactive oxygen species contributed to the hypoxia-mediated increase in DENV replication and to virus-induced hypoxic reprogramming [94] Figure 3.

#### Severe acute respiratory syndrome coronavirus 2

Severe acute respiratory syndrome coronavirus 2 (SARS-CoV-2) is a recently discovered virus that causes a severe respiratory disease known as COVID-19, which has extended throughout the world, eliciting a full-blown pandemic [95]. Importantly, SARS-CoV-2 is known to cause hypoxemia due to the loss of hypoxic vasoconstriction, ventilation/perfusion mismatch, and increased coagulopathy [95]. The angiotensin-converting enzyme 2 (ACE2), a transmembrane protein expressed in the respiratory tract, lungs, heart, arteries, veins, kidney, and intestines has been identified as the cellular receptor required for SARS-CoV-2 infection [96,97]. ACE2 protein levels are decreased upon HIF-1α accumulation in human pulmonary artery smooth muscle cells (hPASMCs) [98], as well as in human embryonic kidney cells overexpressing HIF-1α [99]. Importantly, angiotensin II, which is increased under hypoxic conditions, inhibits the synthesis of its ligand ACE2. Moreover, HIF-1α overexpression in hPASMCs -in which angiotensin II was inhibited- did not result in ACE2 downregulation, thus evidencing a direct relationship between HIF-1α and ACE2 expression [98]. Consistent with this notion, recent reports suggest that populations living in high altitudes (3,000 meters above sea level) such as the Tibetan plateau, El Alto in Bolivia and Ecuador, -which are chronically exposed to hypoxic conditions- appear to display decreased pathology upon SARS-CoV-2 infection [100] Figure 3.

### Negative-sense single-stranded RNA viruses

#### Influenza A virus

**Figure 4.**
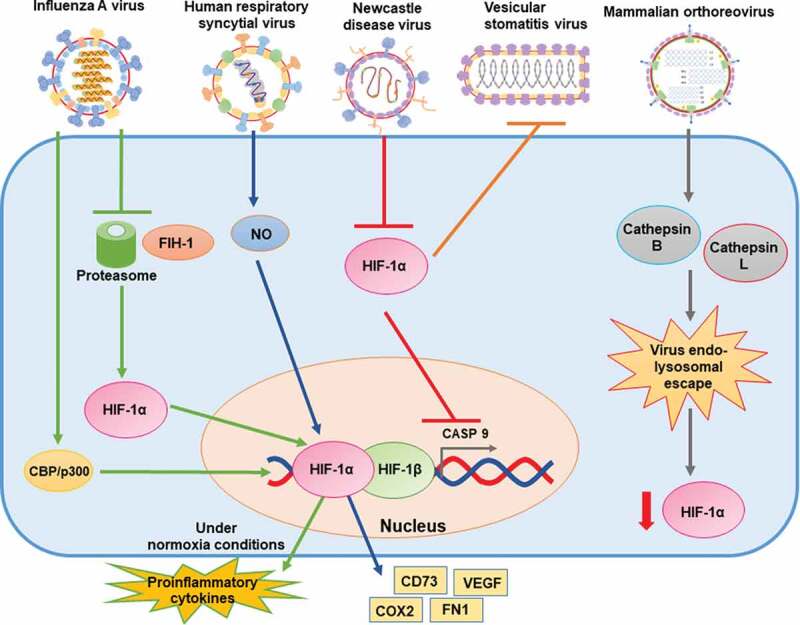
Schematic representation of the effects of negative single-stranded RNA and double-stranded RNA viruses on HIF-1α, and (possible) mechanisms of action

Influenza A virus (IAV) is a zoonotic pathogen constantly challenging the public health systems worldwide, as it has a high mutation rate and kills thousands of people around the world each year due to respiratory disease [101] Some of the most common symptoms are fever, severe headache, sore throat, muscle pain, coughing and fatigue [101]. IAV H1N1 infection of human lung adenocarcinoma epithelial cells stabilizes HIF-1α in normoxic conditions but does not increase HIF-1α mRNA transcription. H1N1 infection did not impair post-translational prolyl hydroxylation or ubiquitination of HIF-1α. Interestingly, HIF-1α stabilization in human lung adenocarcinoma epithelial cells infected with IAV seems to be caused by inhibition of the proteasome and decreases in the expression of the factor inhibiting HIF-1 (FIH-1) [102]. Infection with IAV H1N1 resulted in the translocation of HIF-1α to the nucleus during normoxia. Treatment with 2-methoxyestradiol (2ME2) resulted in inhibition of its nuclear accumulation and decreased mRNA and protein expression levels of TNF-α and IL-6, while also increasing levels of IL-10. Under hypoxic conditions, there was an increase in HIF-1α nuclear accumulation, accompanied by increased levels of TNF-α and IL-6, and decreased levels of IL-10 [103]. Therefore, nuclear translocation of HIF-1α induced by IAV H1N1 infection to the nucleus plays an important role in the production of proinflammatory cytokines. Importantly, recent studies show that deficiency of HIF-1α in a human alveolar type-II epithelial cell line (HAE) reduces glycolysis and enhances AMPKα-ULK1-mediated autophagy, therefore facilitating the replication of this virus [104] Figure 4.

From left to right: Influenza A virus (IAV) stabilizes HIF-1α and promotes its translocation to the nucleus by inhibiting proteasome degradation and decreasing the expression of FIH-1; Human respiratory syncytial virus (hRSV) increases the expression and stabilization of HIF-1α and NO released from infected cells affects these processes; Newcastle disease virus (NDV) inhibits HIF-1α accumulation in a post-translational manner; HIF-1α increases cell resistance to Vesicular stomatitis virus (VSV) infection. Mammalian orthoreovirus (MRV) downregulates HIF-1α likely through endo-lysosomal escape of viral genome into the cytoplasm.

#### Human respiratory syncytial virus

Human respiratory syncytial virus (hRSV) is a respiratory pathogen that accounts for most acute lower respiratory tract infections among pediatric patients and leads to considerable economic burdens for health systems around the globe [105]. hRSV symptoms vary from rhinorrhea, congestion and low grade fever, to bronchiolitis, pneumonia and sever alveolitis [105]. Pulmonary epithelial cells infected with hRSV display activated HIF-1α. An 8-fold increase in HIF-1α protein expression during hRSV infection is reported in HAE cells, and the expression of HIF-1α target genes, such as VEGF, CD73, FN1, and COX2 was also detected [106]. The activation of HIF-1α was suppressed upon UV-inactivation of the virus, suggesting that viral gene expression is required for these effects. Partial oxygen pressure measurements in the supernatants of infected cells showed no changes against controls, suggesting that activation of HIF-1α is independent of oxygen levels in HAE cells infected with this virus [106]. Noteworthy, NO released from cells infected with hRSV may influence HIF-1α expression, as the addition of an inhibitor of NO blocked the expression of HIF-1α in hRSV-infected human bronchial epithelial cells. This treatment also inhibited VEGF production, which has been previously demonstrated to play a role in changing the permeability of bronchial epithelial monolayers [107] Figure 4.

#### Newcastle disease virus

Newcastle disease virus (NDV) is an avian pathogen that elicits high morbidity and mortality in poultry, thus generating high economic burdens to this industry [108]. Depending on the virulence, NDV has been divided into four groups; velogenic viscerotropic (lethal), velogenic nerotropic (high mortality), mesogenic (low mortality) and lentogenic (asymptomatic), each one is characterized by different symptoms [109]. The infection of several cell lines with NDV inhibits HIF-1α protein accumulation and, consequently, decreases the transcription of the HIF-1α target genes, particularly the carbonic anhydrase 9 gene, which encodes the CAIX protein [110]. This protein plays a critical role in primary cancer development, progression, and metastatic disease [111]. Accumulation of HIF-1α was due to post-translational events and decreased upon inhibition of the proteasome, implicating that NDV likely downregulates HIF-1α through the proteasomal pathway. Interestingly, this pathway appeared to be independent of the p53 and VHL host proteins, implicated in regulating the stabilization of HIF-1α [110] Figure 4.

#### Vesicular stomatitis virus

Vesicular stomatitis virus (VSV) is a zoonotic pathogen that affects a wide range of species, from arthropods to humans [112]. Humans’ infection leads to flu-like symptoms without severe illness; however, this virus causes considerable economic losses when infecting cattle, in which symptoms include vesicular lesions of the gums, tongue, naso-oral mucosa, teats and coronary bands [112]. Interestingly, renal carcinoma cells devoid of VHL, which is known to play an important role in the regulation of HIF-1α, conferred cells enhanced resistance to VSV-mediated cytotoxicity. Consistently, hypoxic conditions also promoted resistance to the infection by VSV, as determined by the assessment of live cells. This effect may be explained by HIF-1α-mediated induction of genes with antiviral effects, such as IFN-β. In contrast, the inhibition of HIF-1α enhanced the infection of cells with this virus [113] Figure 4.

### Double stranded RNA viruses

#### *Mammalian* orthoreovirus

Mammalian orthoreovirus (MRV, reovirus) is a highly prevalent benign human virus. Infections with this pathogen usually cause subclinical manifestations and are unnoticed [114]. Three reovirus serotypes are known: type 1 Lang (T1L), type 2 Jones (T2J), and serotype 3 which is divided into the two prototypes type 3 Abeney (T3A) and type 3 Dearing (T3D) [115]. Infection of reovirus-permissive tumor cells and reovirus-resistant tumor cells with reovirus has been shown to significantly downregulate HIF-1α protein levels, which also occurred with UV-inactivated virus, indicating that downregulation of HIF-1α was independent of virus replication [115]. Furthermore, transfection of reovirus genome into human tumor cell lines also downregulated HIF-1α protein levels, even in the presence of polyinosinic-polycytidylic acid (polyI:C), which is a synthetic double-stranded RNA analogue that acts as a pathogen-associated molecular pattern (PAMP), indicating that reovirus RNA plays an important role in this reduction [115]. Interestingly, this effect was inhibited when cells were pretreated with inhibitors of cathepsins B and L, suggesting that endo-lysosomal escape of the reovirus genome into the cytoplasm is crucial for HIF-1α downregulation [115]. Another study also showed that reovirus infection downregulates the expression of HIF-1α-target genes in subcutaneous tumors. However, UV-inactivated virus did not downregulate HIF-1α protein levels in these cells [116] Figure 4.

### Viruses that replicate through DNA intermediates

#### Hepatitis B virus

**Figure 5.**
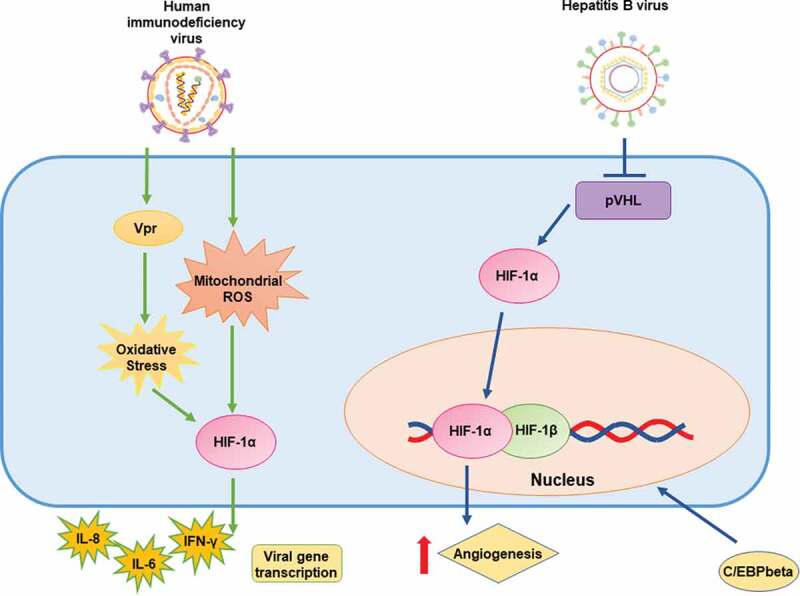
Schematic representation of the effects of retrotranscribing viruses on HIF-1α and (possible) mechanisms of action

Hepatitis B virus (HBV) also causes severe hepatic illness in humans, which is chronic and is estimated to affect approximately 250 million people worldwide [117]. The hepatitis B virus X protein (HBx) is involved in hepatocellular carcinoma [118], and likely in the regulation of viral genes by acting on viral promoters [119]. Studies focusing on the HBx protein have reported increases both in the transcriptional activity and protein levels of HIF-1α in normoxic and hypoxic conditions [118]. This increases also stimulated angiogenesis. Interestingly, HBx inhibited pVHL binding to HIF-1α, preventing the degradation of this transcription factor [118]. Stabilization of HIF-1α by HBx induced the nuclear translocation of C/EBPβ and caused induction of the activity of the transporter of multidrug resistance 1 gene (MDR1) [120] Figure 5.

From left to right: Human immunodeficiency virus (HIV) promotes HIF-1α expression and stabilization via Vpr protein and mitochondrial ROS; Hepatitis B virus (HBV) increases the transcriptional activity and protein levels of HIF-1α. The HBV HBx protein may inhibit pVHL binding to HIF-1α.

#### Human immunodeficiency virus

Human immunodeficiency virus (HIV) causes acquired immunodeficiency syndrome (AIDS) by inducing cell death of T CD4^+^ lymphocytes [121]. The viral protein Vpr promotes HIF-1α expression by activating cellular oxidative stress, which induces HIF-1α protein accumulation and, consequently, stimulates viral gene transcription [122, 123]. Importantly, the HIV promoter has been reported to be positively modulated not only by Vpr but also by HIF-1α through the GC-rich binding domain in the long terminal repeat (LTR) [122]. There is also evidence suggesting that cytosolic double-stranded DNA generated during the viral replication cycle of HIV in CD4^+^ T cells induces mitochondrial ROS-dependent HIF-1α stabilization, which enhances viral replication. Stabilization of HIF-1α promoted the release of extracellular vesicles, which induced the secretion of IFN-γ by bystander CD4^+^ T cells, and the secretion of IL-6 and IL-1β by bystander macrophages [124]. These results suggest that the stabilization of HIF-1α in the context of HIV infection triggers the secretion of inﬂammatory cytokines Figure 5.

**Figure 6.**
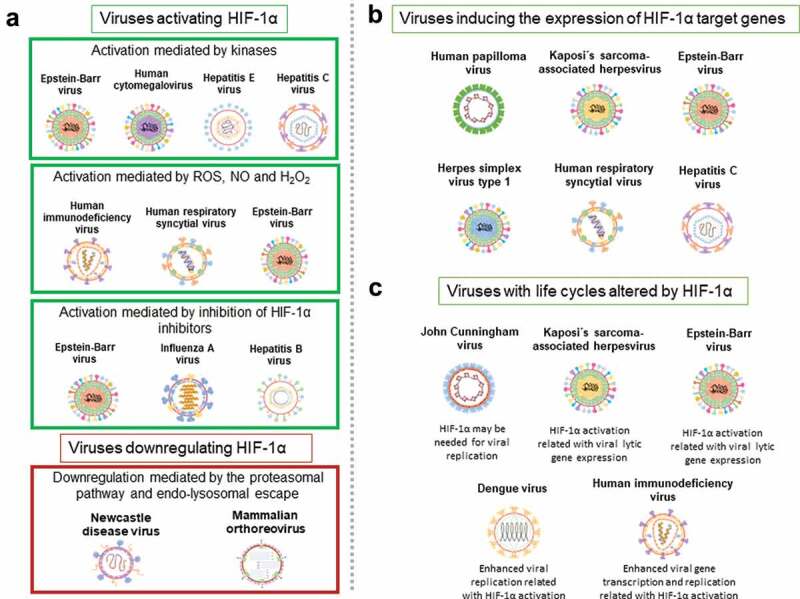
Common features between different viruses and HIF-1α. A

Taken together, while some of the viruses described above downregulate HIF-1 expression and the expression of HIF-1-targeted genes, other viruses activate this factor. Nevertheless, there are some similarities that exist between different viruses in how they affect HIF-1. These aspects are summarized in Figure 6.

Viruses activating or downregulating HIF-1α. Viruses activating HIF-1α via kinases: EBV, HCMV, HEV and HCV. Viruses activating HIF-1α via ROS, NO and H_2_O_2_: HIV, hRSV and EBV. Viruses activating HIF-1α via the inhibition of HIF-1α inhibitors: EBV, IAV and HBV. Viruses downregulating HIF-1α via the proteasomal pathway and by endo-lysosomal escape: NDV and MRV. **B** Viruses inducing the expression of HIF-1α target genes: HPV, KSHV, EBV, HSV-1, hRSV and HCV. **C** Viruses with life cycles altered by HIF-1α: JCV, KSHV, EBV, DENV and HIV.

## HIF-1α as a therapeutic target

HIF-1α is involved in numerous viral infection processes, and hypoxia signaling has been described in a variety of diseases, such as heart conditions (i.e., ischemic heart disease), lung and kidney injuries (i.e., acute lung injury and acute kidney injury), liver steatohepatitis, cancer, and infectious diseases [29, 125, 126].

Therefore, this transcription factor has been considered as a potential target for the development of new therapies [125, 126]. However, it is essential to note that HIF-1 protects the organism during hypoxic conditions, and thus, its inhibition may have detrimental effects for the host [126]. HIF-1 is induced in normoxic conditions in several infectious and non-infectious situations and contributes to the pathophysiology of numerous diseases. Therefore either, the activation or inhibition of HIF-1α may contribute to more favorable outcomes depending on the particular disease.

In the context of organ protection, there are several studies in which HIF-1α was targeted for activation in different tissues with favorable results (e.g., heart, lung, liver, and kidney) [127]. For instance, HIF-1 activity has been shown to be induced after tissue hypoxia due to reduced tissue perfusion, with the consequent transcription of genes encoding for angiogenic factors such as EPO, ANGPT1 and VEGF [128]. These factors play important roles in ischemic heart diseases because they stimulate the remodeling of collateral blood vessels, which leads to increased blood flow in this tissue [128] and play an important role in cardiac adaptation during cardiac hypertrophy [129]. Reportedly, a common method used in clinical trials for stabilizing HIF-1α is ischemic preconditioning/ischemic postconditioning (IPC/IPostC) [126]. There is evidence that HIF-1 is capable of mediating cardioprotection induced by ischemic preconditioning, and by coordinating a balance between glycolytic and oxidative metabolisms [130]. Furthermore, HIF-1 activity has been reported to be important at maintaining O_2_ homeostasis during compensatory myocardial hypertrophy in response to pressure overload, and to play an important role in protecting against pressure overload after heart failure [129, 128]. Consistently, knockdown of endothelial HIF-1α in the heart increased TGF-β signaling, which led to pathological remodeling [131]. However, it is noteworthy that, in some cases contrary effects have been described after HIF-1 overexpression. For example, a study reported that increasing the expression of cardiac HIF-1α resulted in aggravated heart failure [132].

Another strategy considers the use of inhibitors of PHDs, which lead to the activation of HIF-1α. To date, non-specific PHDs inhibitors, such as vadadustat, roxadustat, daprodustat, and molidustat are being tested or have been recently evaluated in clinical trials for the treatment of patients with chronic kidney disease (CKD) [126, 133]. This illness increases systemic oxidative stress and inflammation, activates the renin-angiotensin system, and causes nutritional disorders, which altogether may increase the risk of cardiovascular events [134, 135]. Anemia caused by a reduction in renal erythropoietin (EPO), as a consequence of CKD may also affect cardiac function [135]. Interestingly, the gene encoding EPO is a target of HIF-1, although primarily HIF-2 [136]. Direct administration of molecules that are induced by HIF-1, such as EPO, are also being evaluated for their ability to protect the heart and kidneys from acute injuries, as well as for treating anemia in CKD [126, 133].

Cancer is another disease that has been reported to have a connection with HIF-1. Immunohistochemical analyses of several types of cancer show an overexpression of HIF-1α [137]. Interestingly, this effect is caused by gain of functional mutations in oncogenes, loss of functional mutations in tumor suppressor genes, as well as a result of intratumor hypoxia [138, 139, 140, 137]. Although the overexpression of HIF-1 in head and neck cancers and non-small-cell lung cancer was reported to reduce the mortality of the patients, other studies have failed to reproduce these effects [141, 142, 143, 144, 137]. Indeed, other reports have associated HIF-1α expression with increased mortality for several cancers, such as cervical, breast, endometrial, oropharyngeal and ovarian cancer [145, 146, 147, 148, 149, 137].

Currently, several studies exploring the potential of HIF-1α inhibition for treating cancer are ongoing, as HIF-1α activity increases solid tumor survival, as well as tumor aggressiveness, invasion, and metastasis [150, 152]. In this regard, HIF-1α inhibitors are being assessed both in pre-clinical and clinical trials with two major types of HIF-1 inhibitors: direct or indirect inhibitors, which modulate factors that are up- or downstream of HIF-1-related signaling pathways [125]. Importantly, HIF-1α and hypoxic conditions affect the function of immunosuppressive myeloid-derived suppressor cells (MDSCs) in tumors, and thus the modulation of this factor may have direct effects over these cells [153]. Other studies have reported that HIF-1 plays a role in tumor resistance to chemo- and radiotherapy [125]. Interestingly, a combination of inhibitors of HIF-1α and chemotherapy showed a higher anti-tumor effect than the chemotherapy agent gemcitabine alone [154]. A combination of radiotherapy and HIF-1α inhibitors showed similar results, increasing the tumor’s sensitivity to radiation [155]. The role of HIF-1α in other ailments and infectious diseases (e.g. bacterial) has been extensively reviewed by Semenza and dos Santos [29, 23].

Alternatively, targeting HIF-1 may also help treat pathogenesis associated with inflammation, as HIF-1α regulates essential pathways for maintaining energy homeostasis in myeloid cells [156]. Additionally, inactivation of HIF-1α has been reported to inhibit the motility, invasiveness, and homotypic adhesion of isolated peritoneal macrophages [156]. Given that inhibition of glycolysis prevents inflammatory responses, and glycolytic metabolism-related genes are targeted by HIF-1α activation, the inhibition of this transcription factor may help inhibit inflammatory responses [157]. Therefore, as inflammation is a component or a symptom related to the severity of numerous infectious diseases, inhibiting HIF-1 may have potential therapeutic effects against microbial infections [157].

For instance, this could be the case for the Kaposi’s sarcoma-associated herpesvirus, Epstein-Barr virus, and human respiratory syncytial virus, in which cases the expression of HIF-1 target genes has been reported to have positive effects regarding the pathogenicity of viral infections [77, 69, 107, 76]. Particularly, VEGF has been shown to play essential roles in KSHV and hRSV infections, so inhibition of HIF-1 and, therefore VEGF inhibition, may help overcome either infection or even decrease disease severity.

HIF-1α expression can be suppressed with siRNAs or specific molecules. Interestingly, several studies have used siRNAs to understand the effect of HIF-1 in different contexts [158, 159]. For example, inhibition of HIF-1α with a siRNA promoted a metabolic shift in human pluripotent stem cell-derived cardiomyocytes [158], and in podocytes where the inhibition of this transcription factor decreased the expression of inflammatory cytokines [159]. On the other hand, numerous molecules that inhibit HIF-1α expression have been discovered or synthetized as a therapy for cancer. There are different mechanisms by which these molecules inhibit this transcription factor, such as through the inhibition of mRNA expression (e.g. by using EZN-2968 or EZN-2208), inhibition of protein synthesis (e.g. by using 2-methoxyestradiol or CAY10585), or by promoting its degradation (e.g. by using 17-DMAG or bisphenol A) [160], among others.

Some molecules such as vadadustat, roxadustat, daprodustat, and molidustat, which are PHDs inhibitors, have opposite effects compared to the molecules described above and activate HIF-1α. These molecules may be used for developing antiviral treatments for viruses in which HIF-1 activity may be detrimental, such as VSV, as cells that do not express the von Hippel-Lindau protein are more resistant to VSV-mediated cytotoxicity [113]. Also, this type of drug may be used for treating NDV, because infection with this virus downregulates HIF-1α stabilization, and therefore the activation of this transcription factor may have an antiviral effect [110]. Finally, these types of drugs may be used against SARS-CoV-2, as ACE2 which is crucial for viral infection, decreases upon HIF-1α accumulation [98].

Till date, there are no drugs capable of modulating HIF-1 that are being tested in clinical trials for the control of viral infections. Nevertheless, we foresee this to happen in the short term. Inhibition of HIF-1 should be approached with caution in microbial infections, as studies report uncertain outcomes for infectious diseases when stimulating HIF-1. For example, Okumura *et al*. reported that stabilizing HIF-1α with AKB-4924 -an inhibitor of PHDs- resulted in a positive effect on the innate immune response by enhancing the antibacterial activity of macrophages and keratinocytes against methicillin-sensitive and methicillin-resistant *Staphylococcus aureus*. The same drug stimulated the bacteria-killing capacity of keratinocytes against *Pseudomonas* [161].

## Concluding remarks

Numerous studies carried out till date support that HIF-1 plays relevant roles in the infectious cycles of numerous viruses, as well as the host response to these pathogens. Noteworthy, most viruses studied to date have been shown to positively modulate this pathway by upregulating the stabilization of HIF-1α as a consequence of infection. Importantly, HIF-1 also plays an important role in the modulation of both, the innate and the adaptive immune responses of the host against viral infection.

Although the relationship between HIF-1-related pathways and the modulation of the life-cycles of some viruses have been dilucidated, the precise molecular associations occurring with several other viruses and this host factor remain to be determined. However, some common features exist among different types of viruses Figure 6.

Despite the involvement of HIF-1 in the infection of diverse viruses, there are reportedly no ongoing clinical trials evaluating the potential therapeutic effects of the pharmacological modulation of HIF-1 in viral infections. However, given the increasing amount of evidence regarding the participation of HIF-1 in viral infections, we foresee that additional studies will originate in the short term, assessing the contribution of this host factor over the replication of viruses and viral diseases, as well as the mechanisms of action by which HIF-1 and its related pathways influence viral infection and disease outcomes.
